# Assessing Reporting Quality and Pre-Analytical Standards in Extrachromosomal Circular DNA Studies in Cancer: A Systematic Review

**DOI:** 10.3390/cancers18142196

**Published:** 2026-07-08

**Authors:** Felishia Tian, Sarah Soyeon Oh, Chul S. Hyun, Han Sang Kim, Jae Il Shin

**Affiliations:** 1 College of Literature, Science, and the Arts, University of Michigan, Ann Arbor, MI 48109, USA; tzhen@umich.edu; 2 Institute for Global Engagement & Empowerment, Yonsei University, Seoul 03722, Republic of Korea; sarahoh@yonsei.ac.kr; 3 Division of Digestive Diseases, Department of Medicine, Yale School of Medicine, New Haven, CT 06510, USA; chul.hyun@yale.edu; 4 Yonsei Cancer Center, Division of Medical Oncology, Department of Internal Medicine, Yonsei Institute for Digital Health, Yonsei University College of Medicine, Seoul 03722, Republic of Korea; 5 Department of Pediatrics, Yonsei University College of Medicine, Seoul 03722, Republic of Korea; 6 Severance Underwood Meta-Research Center, Institute of Convergence Science, Yonsei University, Seoul 06273, Republic of Korea

**Keywords:** eccDNA, systematic reviews, guideline, cancer, extrachromosomal circular DNA

## Abstract

**Background/Objectives**: eccDNA is a promising cancer biomarker in liquid biopsy. However, the reliability and reproducibility of eccDNA studies rely on the standardization of pre-analytical handling and processing of eccDNA, as well as transparent methodological reporting. Although evidence-based guidelines for cell-free DNA handling provide clear recommendations for plasma/serum processing, the extent to which eccDNA studies report and adhere to these key procedures remains uncertain. **Methods**: We systematically reviewed 14 studies (2017–2025) assessing eccDNA in plasma or serum from cancer patients. Each study was evaluated against 22 checklist items summarized from the NCI Biospecimen Collection and Processing Guideline, categorized into biospecimen collection, blood processing, and eccDNA processing. Items were classified as “reported,” “not reported,” or “deviated from NCI guideline,” and missing/deviation rates were calculated. **Results**: Publication was largely post-guideline (13/14 after 2020). Reporting gaps were widespread. Items with 100% missing were venipuncture site (14/14), freeze–thaw cycles (14/14), and fitness for downstream analysis/pre-assay QC (14/14). Other frequently missing elements included blood volume (10/14; 71%), collection tube type (7/14; 50%), date/time from draw to processing (11/14; 79%), second centrifugation parameters (8/14; 57%), storage temperature before freezing (6/14; 43%), frozen storage duration (13/14; 93%), and post-thaw handling (13/14; 93%). By contrast, the extraction method was consistently reported (0% missing), and most reported quantification (21% missing). Deviations from the NCI guideline were uncommon when items were reported, such as a blood processing delay (2/14; 14%) and the use of circulating nucleic acid kits for eccDNA extraction (1/14; 7%). **Conclusions**: Inconsistent reporting of pre-analytical procedures limits the reproducibility, transparency, and clinical translation of eccDNA research. We propose a concise reporting checklist informed by the NCI guideline. By focusing on the most frequently underreported aspects of key pre-analytical eccDNA procedures, the checklist provides researchers with an efficient and succinct methodological reporting framework that will enhance transparency and promote standardization in future eccDNA studies.

## Simple Summary

Extrachromosomal circular DNA (eccDNA) shows potential as a cancer detection biomarker in liquid biopsy. We systematically reviewed 14 studies published between 2017 and 2025 that used eccDNA in plasma or serum from cancer patients, evaluating their methods against 22 checklist items summarized from the United States National Cancer Institute (NCI) “Cell-free DNA: Biospecimen Collection and Processing” guideline. Reporting gaps were significant, particularly in procedural details for eccDNA isolation, which limits the reproducibility and validity of eccDNA research. Based on our findings, we propose a concise reporting checklist for eccDNA isolation methods aligned with the NCI guideline to improve transparency and method standardization in future eccDNA studies.

## 1. Introduction

Extrachromosomal circular DNA (eccDNA) is a form of circular DNA derived from chromosomal DNA but existing independently of chromosomes [1,2]. Ranging in size from <100 base pairs to several megabases, eccDNA is detectable in human circulation as a component of cell-free DNA (cfDNA) [3,4]. Tumors release eccDNA into the bloodstream, and circulating levels correlate with disease burden [2,3]. For example, pre-operative plasma often contains higher eccDNA concentrations than matched post-resection samples [3]. Malignant tumors typically harbor more eccDNA than benign lesions, and elevated eccDNA levels in cancer patients have been associated with poorer survival [2]. Functionally, eccDNA can promote oncogene amplification by acting as a transcriptional regulatory element, such as an enhancer or promoter, and may also participate in tumor-associated pro-inflammatory responses [1]. Given the critical role of the immune system in early cancer development, eccDNA–immune interactions may provide valuable opportunities for early detection. Collectively, these findings highlight the potential of eccDNA as a biomarker for cancer detection, diagnosis, prognosis, and disease monitoring [4].

Liquid biopsy has gained traction in oncology due to its non-invasive, cost-effective nature and capacity for real-time monitoring through repeat sampling [1]. However, the small quantities of eccDNA present in biofluids pose challenges for detection, and current enrichment methods require improved sensitivity [3,4]. Because enrichment and downstream analytical methods can substantially affect results, methodological standardization is essential to ensure reliability, comparability, and reproducibility [4]. In particular, minimizing genomic DNA contamination can increase the proportion of informative reads and, consequently, reduce sequencing costs [4].

Many eccDNA studies follow the three-step eccDNA purification method, which involves the isolation of crude circular DNA, linearization of mitochondrial DNA, digestion of linear DNA, and purification of circular DNA from residual linear DNA [5]. Minor modifications at any step can yield divergent results, reflecting the heterogeneity and low abundance of eccDNA in patient samples [6]. Additional variability arises from pre-analytical factors, such as biospecimen collection, processing, and storage [7]. Although many institutions implement standard operating procedures, these often differ across sites, and critical pre-analytical details, especially for blood collection and handling, remain underreported [7]. Notably, there are no validated eccDNA-specific reporting guidelines for plasma or serum, raising concerns about reproducibility and translational readiness. In the absence of such guidelines, existing evidence-based recommendations for biospecimen collection and processing may provide a helpful guiding framework for identifying key pre-analytical details that should be transparently reported in eccDNA studies.

To address inconsistencies in pre-analytical practices for cfDNA more broadly, the Biorepositories and Biospecimen Research Branch of the U.S. National Cancer Institute (NCI) issued an evidence-based guideline in January 2020 as part of its Biospecimen Evidence-Based Practices series: “Cell-free DNA: Biospecimen Collection and Processing” [7]. The guidance provides detailed, literature-supported recommendations from biospecimen collection through cfDNA extraction and quantification, delineating optimal practices and acceptable alternatives to accommodate institutional variability.

In this study, we systematically reviewed 14 studies published between 2017 and 2025 that analyzed eccDNA from the plasma or serum of cancer patients. Each study was assessed for deviations from the NCI guideline recommendations and for the lack of reporting of key pre-analytical methodological variables identified in the guideline. Our goal was to determine the extent to which these variables were reported and to identify reporting gaps that could hinder the reproducibility and clinical translation of eccDNA research. For the purposes of this review, key pre-analytical variables described in the NCI guideline were adapted into a reporting assessment checklist; the NCI guideline itself is not a formal methodological reporting guideline.

## 2. Materials and Methods

### 2.1. Eligibility Criteria

We included primary research studies published between January 2014 and July 2025 that reported eccDNA from plasma or serum in cancer patients. Eligible studies were required to describe at least two of the following: (1) blood collection, (2) blood processing, and (3) eccDNA isolation procedures. We excluded review articles, conference abstracts without full data, non-human studies, studies not involving eccDNA, studies in non-cancer patients, and studies that did not extract eccDNA from plasma or serum. In the absence of a standardized methodological reporting checklist for eccDNA studies, included articles were evaluated using recommendations described in the *NCI Cell-free DNA: Biospecimen Collection and Processing* guideline as a reference framework [7]. This guideline provides rigorously supported, evidence-based guidance for every stage of the workflow, spanning from biospecimen collection preparation to cfDNA quantification and quality evaluation [7]. These procedural guidelines emphasize best practices for specimen handling in cfDNA analyses while also allowing validated alternative approaches to account for institutional variability in equipment and resources without sacrificing sample integrity [7]. Although the search window began in 2014, the earliest eligible study was published in 2017.

### 2.2. Information Sources

We systematically searched PubMed, last searched on 25 June 2025, and Google Scholar, which was last searched on 1 July 2025. The reference lists of included articles and relevant reviews were also screened for additional eligible studies (Table 1 and Supplementary Table S1).

### 2.3. Search Strategy

The search string used for both databases was (“extrachromosomal circular DNA” OR “eccDNA” OR “circular ecDNA” OR “circular extrachromosomal DNA” OR “circular DNA”) AND “cancer” AND (“plasma” OR “serum” OR “liquid biopsy”). We retrieved the full texts of potentially eligible articles and assessed them against the inclusion and exclusion criteria.

### 2.4. Study Selection

**Figure 1:**
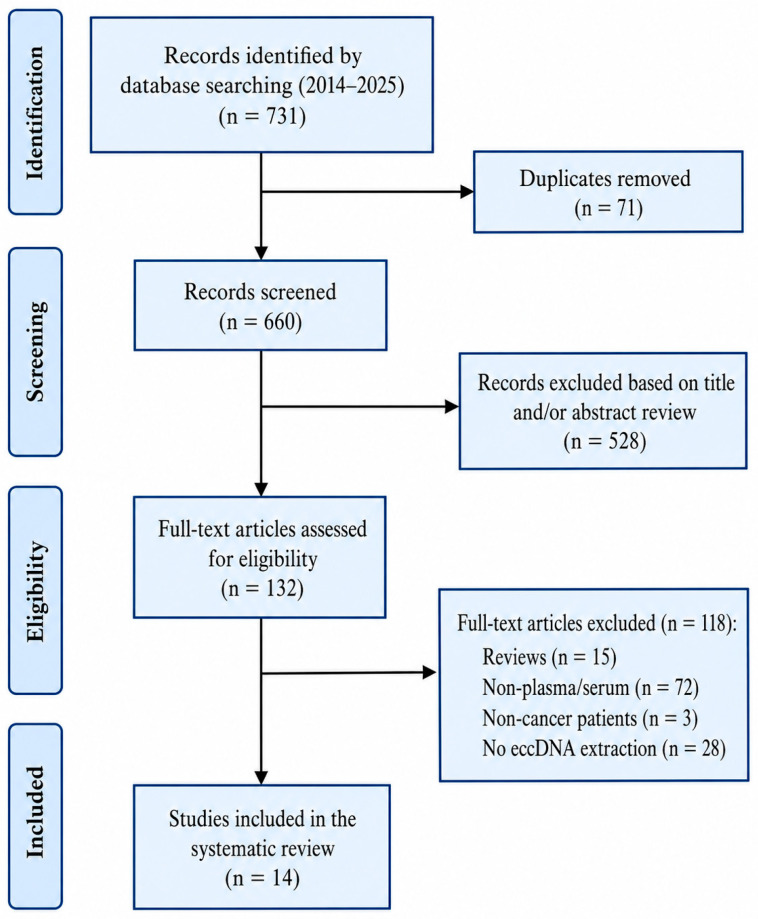
Study selection flow diagram.

Two reviewers independently screened all retrieved records by title and abstract, followed by a full-text review of potentially eligible studies. Discrepancies were resolved by consensus. A total of 14 studies met the inclusion criteria: 11 full-length journal articles, two letters, and one preprint. The study selection process is summarized in the PRISMA flow diagram (Figure 1). This review was performed in accordance with the PRISMA (Preferred Reporting Items for Systematic Reviews and Meta-Analyses) 2020 guidelines and has not been registered. A separate review protocol was not prepared. The PRISMA systematic review checklist for this review can be found in Supplementary Table S2. Data extraction and checklist coding were performed by one reviewer (F.T.) and verified by a second reviewer (H.S.K.); disagreements were resolved by consensus.

### 2.5. Data Items

For each included study, we extracted general study characteristics, including the publication year, cancer type, patient sample size, and biospecimen type (plasma or serum). We additionally examined the methodological details of these studies, categorized into three domains based on the *NCI Cell-free DNA: Biospecimen Collection and Processing* guideline [7].

(A) Biospecimen Collection

We evaluated each study’s completeness in reporting patient information, including age, gender, clinical diagnosis, fasting status, and treatment status at the time of sampling. We also looked for reporting of collection considerations, such as collection tube type and the volume of blood drawn from patients. Furthermore, we assessed the reporting of details during the actual blood collection process, including the date/time of collection, the venipuncture site, and the type of needle used.

(B) Blood Processing

We evaluated whether each study reported any delays between blood collection and processing. We examined the reporting of plasma/serum separation procedures during processing, including centrifugation speed, temperature, and the number of centrifugations performed. We also assessed the reporting of interim storage conditions, including storage duration, temperature, and the number of freeze–thaw cycles.

(C) eccDNA Processing

We examined the reporting of the eccDNA isolation process, including the materials and protocols used for extraction, digestion, and purification. Additionally, we assessed the reporting of methods and technologies used for eccDNA quantification following isolation. We also looked for the reporting of any suitability assessment of eccDNA for downstream analysis. Lastly, we evaluated whether studies validated their analytical assays for accuracy, precision, specificity, and sensitivity.

### 2.6. Risk of Bias and Quality Assessment

We developed a 22-item methodological reporting checklist summarized from pre-analytical variables described in the NCI guideline that were applicable to plasma- and serum-based eccDNA workflows. The items were directly organized based on the NCI guideline recommendations because the pre-analytical variables (such as methods of blood sample collection, processing times, centrifugation parameters, storage conditions, DNA isolation processes, and DNA quality evaluation) are thought to impact the integrity of cfDNA, which includes eccDNA. No additional reporting domains were included or excluded; instead, the NCI guideline was transformed into an assessment framework that was systematically applied to included studies in this review. Each item was coded as “reported,” “not reported,” or “deviated from NCI guideline recommendation.” Missing and deviation rates were calculated for each item across all studies: missing rate = (# of studies that have not reported an item) ÷ (total # of studies); deviation rate = (# of studies that have deviated from the NCI recommendation) ÷ (total # of studies). Because the objective of this review was to assess the completeness of pre-analytical methodological reporting rather than diagnostic accuracy or effect estimates, formal risk-of-bias tools such as QUADAS-2 [22] were not applied. QUADAS-2 appraises the risk of bias and applicability in diagnostic accuracy studies across four domains—patient selection, index test, reference standard, and flow and timing—which are designed to evaluate the validity of diagnostic performance estimates. Although several included studies pursued diagnostic biomarker aims, our unit of assessment was the reporting of pre-analytical variables (collection, processing, storage, and extraction) rather than diagnostic test accuracy; consequently, the QUADAS-2 domains do not map onto the reporting items evaluated here. A checklist-based reporting assessment was therefore better suited to the aims of this review.

To provide an overall summary of reporting quality, each study was additionally classified according to the proportion of the 22 checklist items reported into high (≥50% reported), intermediate (30–49%), and low (<30%) reporting-completeness categories.

### 2.7. Data Synthesis

Descriptive statistics were used to summarize the proportion of studies reporting each checklist item, as well as the missing and deviation rates within each domain. Horizontal bar graphs summarize the missing and deviation rates for each item. Descriptive statistics and graph generation were performed using Excel. No meta-analysis was performed due to methodological heterogeneity and the qualitative focus of this review.

## 3. Results

### 3.1. Study Characteristics

We identified 14 studies between 2017 and 2025 that quantified or characterized eccDNA in plasma or serum from patients across diverse malignancies, including lung, colorectal (CRC), pancreatic, cholangiocarcinoma, thyroid, breast, prostate, hepatocellular (HCC), renal cell carcinoma, and multiple myeloma (Table 1). Sample sizes ranged from 6 to 252 participants. Plasma was the most common matrix; three studies used serum, and six incorporated matched tumor tissue (breast, prostate, HCC, CRC, lung, and thyroid). One study included bile alongside plasma, and one included urine. Many studies enrolled healthy controls or benign disease comparators (e.g., prostatitis or nodular thyroid goiter), enabling case–control comparisons. Of note, 13 of the 14 studies were published after 2020, following the release of the NCI cfDNA collection/processing guideline, thereby situating most of the evidence within the contemporary pre-analytical framework (Table 1).

### 3.2. Completeness of Reporting for Guideline-Recommended Items

**Figure 2:**
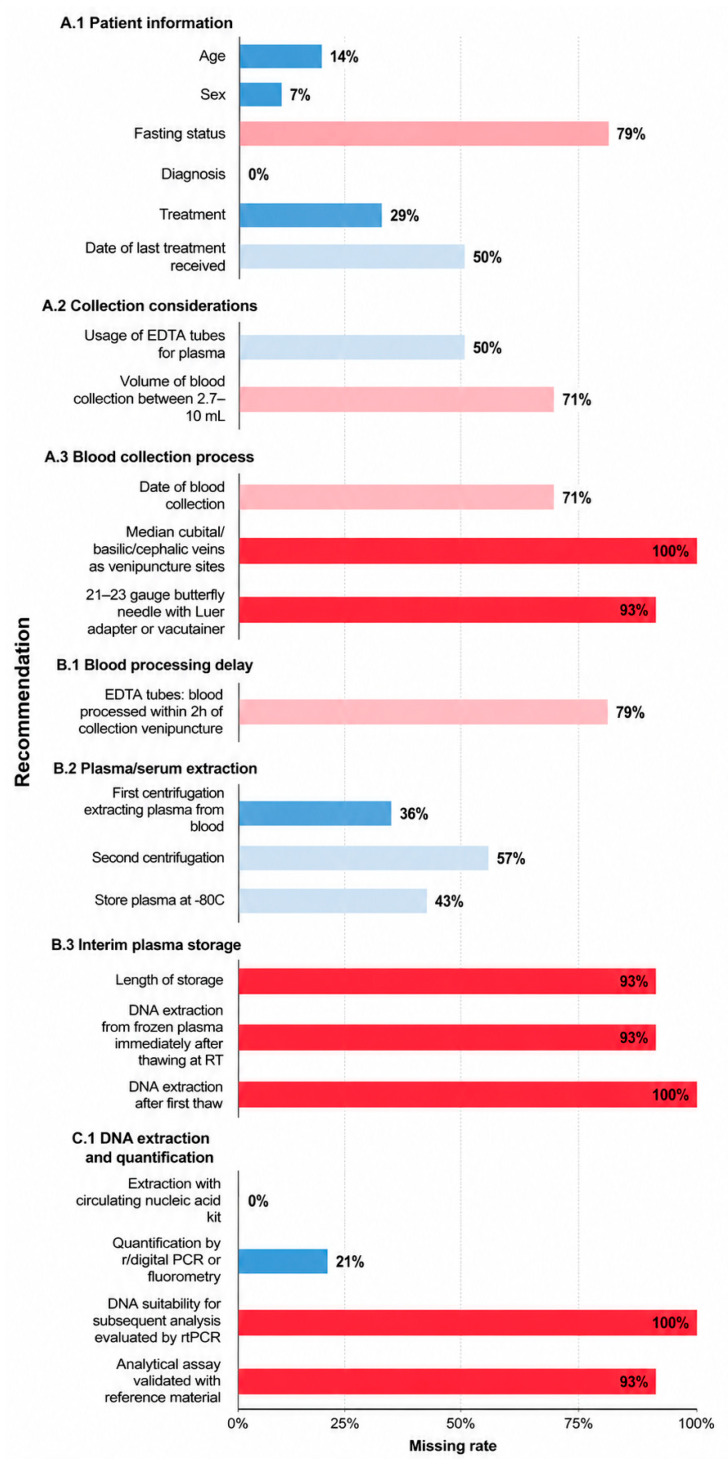
Missing rate of information recommended by the guideline. Bar colors visually represent the magnitude of the missing rate, ranging from blue for lower missing rates to red for higher missing rates.

Across all guideline-recommended items, the missing rate (proportion of studies not reporting a given item) ranged from 0% to 100%, with patient-level descriptors generally better reported than pre-analytical and validation details (Figure 2).

Regarding biospecimen collection, studies consistently reported patient diagnosis (0% missing). Basic patient demographics were usually provided, although age was absent in 2/14 (14%) and sex in 1/14 (7%) of the reports. By contrast, fasting status was rarely documented, with 11/14 (79%) of studies missing it. Reporting of key blood-draw parameters was similarly limited. The collection tube type was not specified in 7/14 (50%) of studies, and blood volume was not provided in 10/14 (71%) of studies. Procedural details were especially sparse. The date of collection was unreported in 10/14 (71%) of studies, the venipuncture site in 14/14 (100%) of studies, and the needle type in 13/14 (93%) of studies. Regarding blood processing, most studies did not state the interval between collection and processing, with this item missing in 11/14 (79%) of studies. Separation parameters were variably reported. First centrifugation conditions were absent in 5/14 (36%) of studies, and second centrifugation conditions in 8/14 (57%) of studies. Details on short-term handling were incomplete, as the storage temperature for plasma/serum before long-term freezing went unreported in 6/14 (43%) of studies. Information on frozen storage was particularly limited: the duration of storage and whether DNA extraction occurred immediately after thawing were each missing in 13/14 (93%) of studies, and freeze–thaw cycles were not reported by any study (14/14; 100%).

For eccDNA processing and analytical validation, all studies described the extraction method (0% missing), and most reported quantification, although 3/14 (21%) did not. None of the studies documented fitness for downstream analyses (e.g., pre-assay QC metrics), resulting in 14/14 (100%) missing. Likewise, assay validation, including accuracy, precision, sensitivity, and specificity, was rarely addressed, with 13/14 (93%) not reporting these metrics.

**Figure 3:**
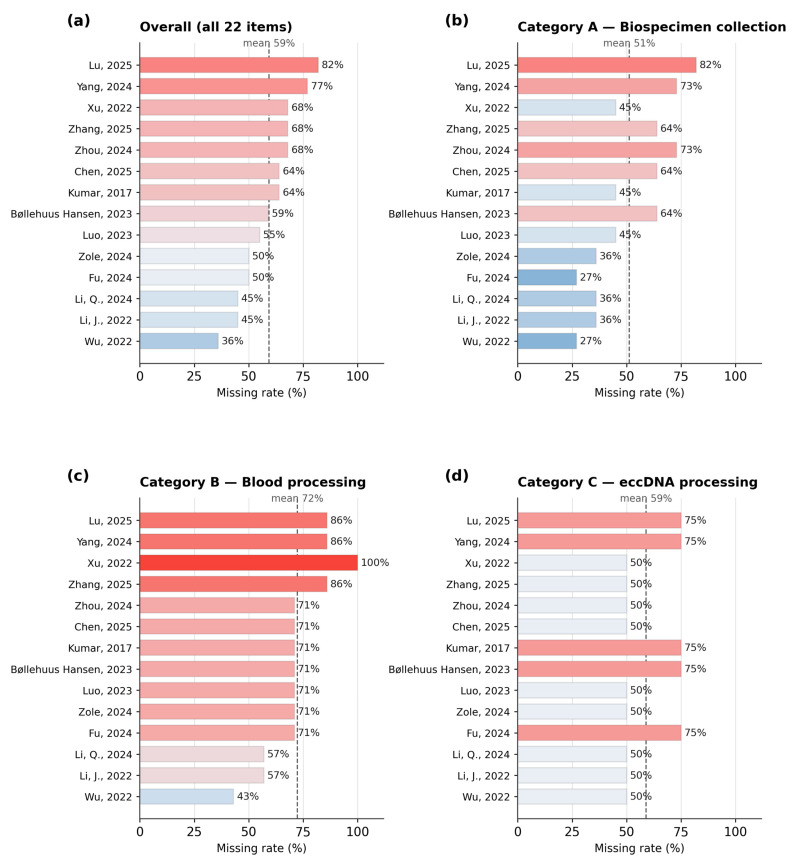
Study-level reporting gaps, expressed as the missing rate (100%—percentage of recommended items reported) for each of the 14 included studies: Kumar et al. [8], Li, J. et al. [9], Wu et al. [10], Xu et al. [11], Bøllehuus Hansen et al. [12], Luo et al. [13], Fu et al. [14], Li, Q. et al. [15], Yang et al. [16], Zhou et al. [17], Zole et al. [18], Chen et al. [19], Lu et al. [20], and Zhang et al. [21]. The panels show (**a**) all 22 checklist items combined and the three pre-analytical domains: (**b**) Category A, biospecimen collection; (**c**) Category B, blood processing; and (**d**) Category C, eccDNA processing. Studies are ordered by overall missing rate, and bar color scales with the missing rate (blue, lower; red, higher). Dashed lines denote the mean missing rate within each panel. Reporting was least complete for blood processing (Category B; mean 72% missing), and no study reported more than 64% of all items.

Overall, the average number of items reported per study was 9 out of 22 (41%). Across the 14 studies, the percentage ranged from 14/22 (64%) to 4/22 (18%). Using this classification, 5/14 studies were categorized as high (≥50% of items reported), 7/14 as intermediate (30–49%), and 2/14 as low (<30%) in reporting completeness (Supplementary Table S1). Notably, no study reported more than 64% of the items, indicating that even the most complete reports omitted a substantial share of recommended pre-analytical variables (Figure 3).

### 3.3. Deviation from Reporting Guideline Recommendations

Among the reported items, deviations from the NCI guideline’s recommended practices were infrequent (Supplementary Figure S1). Non-zero deviations were observed only for delays in blood processing, including reported practices deviating in 2/14 (14%) of studies, and for the use of circulating nucleic acid kits for eccDNA extraction, which deviated in 1/14 (7%). For all other reported items, studies aligned with the guideline recommendations (0% deviation). Since deviation cannot be determined for unreported items, the actual frequency of non-conformant practice also cannot be reliably assessed. The item-level checklist classifying each element as reported, missing, or deviated is provided in Supplementary Table S1.

Taken together, the most consistent reporting gaps clustered around the blood collection process (date/site/needle/volume/tube), interim and frozen storage conditions (temperature, duration, freeze–thaw history, post-thaw handling), and analytical validation. These omissions hinder the assessment of biospecimen integrity and cross-study comparability. Of note, limitations are especially consequential for low-abundance targets such as eccDNA.

## 4. Discussion

In this review, we systematically assessed studies investigating eccDNA in cancer patient plasma and serum, focusing on their adherence to the pre-analytical methodological details outlined in the *NCI Cell-free DNA: Biospecimen Collection and Processing* guideline [7]. Our findings reveal that reporting completeness was highly variable across studies. Although basic patient information, such as age and sex, was consistently provided, crucial pre-analytical details, including the venipuncture site, blood collection volume, duration of biospecimen storage, and validation of analytical assays, were almost uniformly omitted. In contrast, deviations from NCI-recommended practices were relatively uncommon and limited to two items (blood processing delay and DNA extraction). However, it is not possible to determine the true frequency of deviations in these studies due to the large percentage of omitted pre-analytical details, further emphasizing the problem of underreporting as the primary limitation in the current literature.

Several domains emerged as critical gaps in reporting. First, the blood collection process was rarely described in sufficient detail. Information on the date and volume of collection, venipuncture site, and needle choice was consistently absent. Beyond limiting reproducibility, inadequate reporting in this area also raises concerns regarding biospecimen quality, as suboptimal collection practices can contribute to hemolysis and genomic DNA contamination, potentially obscuring eccDNA detection, which is already challenging due to its low abundance. Second, interim plasma storage details were frequently omitted, including storage duration, the number of freeze–thaw cycles, and whether DNA extraction was performed immediately after thawing. Repeated freeze–thaw cycles have been shown to alter cfDNA fragment size distributions significantly, and the NCI guideline recommends minimizing freeze–thaw cycles to preserve nucleic acid integrity. Without such details, it is difficult to assess the reliability of the reported results or to reproduce comparable storage conditions. Third, the qualification of the eccDNA samples and the validation of the analytical assays were largely absent. None of the reviewed studies reported evaluating sample suitability for downstream analyses, and the majority failed to report using reference materials to benchmark assay sensitivity and precision.

The issue of poor reporting of methodological details can also serve as a source of bias when analyzing eccDNA as a cancer biomarker. If essential pre-analytical variables are poorly described, it is difficult to understand whether any observed differences in eccDNA yield, fragment composition, or biomarker performance arise from biological variance or result from variations in sample handling and preparation procedures. It is also harder to achieve consistency in findings and estimates between different studies due to this issue, which can further hinder attempts to validate study results. Incomplete reporting of methodological details also prevents the process of reproducing research protocols, investigating technical biases, and testing the generalizability of study results. A lack of reporting detail can slow down the process of creating standardized methods and the generation of evidence to support the wider use of eccDNA in clinical settings.

**Figure 4:**
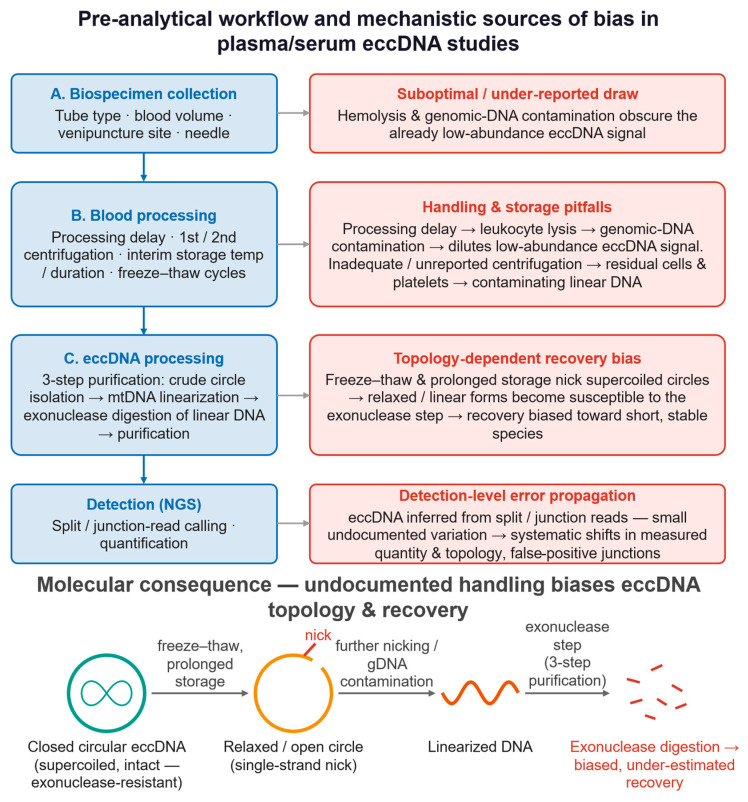
Pre-analytical workflow for plasma/serum eccDNA studies (left) and the mechanistic sources of bias introduced when individual steps are not standardized or reported (right). Across biospecimen collection (A), blood processing (B), and eccDNA processing (C), inadequate or undocumented handling promotes genomic DNA contamination and alters the structure of circular molecules. The lower panel summarizes the resulting topology cascade: intact, exonuclease-resistant supercoiled eccDNA acquires single-strand nicks during freeze–thaw cycles and prolonged storage, relaxing and ultimately linearizing the circles so that they become susceptible to the exonuclease step of purification. Because eccDNA is quantified from split/junction reads, these undocumented changes propagate into systematic differences in measured abundance, fragment topology, and false-positive junction calls. Blue boxes denote workflow steps; red boxes denote the associated pre-analytical pitfalls.

Mechanistically, the pre-analytical variables identified as poorly reported are precisely those most likely to distort eccDNA topology, abundance, and sequencing fidelity. eccDNA enrichment typically relies on exonuclease-based digestion of linear DNA within a multi-step purification of closed-circular molecules [5], a workflow that depends on the structural integrity of the circles. Repeated freeze–thaw cycles and prolonged storage can introduce single-strand nicks that convert supercoiled circles into relaxed or linearized forms, rendering them susceptible to exonuclease digestion and biasing recovery toward shorter, more stable species; the same processes shift cfDNA fragment size distributions and can therefore alter the apparent eccDNA size profile [6,7]. Delayed processing and the absence of stabilizing collection tubes promote leukocyte lysis and genomic DNA contamination, which dilute the low-abundance eccDNA signal, lower the proportion of informative junction-spanning reads, and inflate background during sequencing [7]. Suboptimal or unreported centrifugation can leave residual cells and platelets that contribute additional contaminating linear DNA [23]. Because eccDNA is present at very low abundance and is identified bioinformatically through split/junction reads, even modest, undocumented variation in these steps can translate into systematic differences in measured eccDNA quantity, fragment topology, and false-positive junction calls, undermining quantitative interpretation and cross-study comparability (Figure 4).

Beyond general reproducibility, incomplete pre-analytical reporting has tumor-type-specific consequences for eccDNA biomarker development. The studies reviewed here span malignancies with markedly different circulating tumor burdens and eccDNA yields—from hematological cancers such as multiple myeloma to solid tumors including lung, colorectal, prostate, renal, and thyroid carcinomas. Because eccDNA abundance and fragment characteristics differ across tumor types and disease stages, undocumented variation in collection tubes, processing delay, centrifugation, and freeze–thaw history makes it difficult to determine whether between-study differences reflect genuine tumor biology or divergent specimen handling. This ambiguity directly affects biomarker discovery (identifying tumor-specific eccDNA signatures), diagnostic performance (establishing reproducible thresholds and limits of detection), and cross-tumor comparability (benchmarking eccDNA against established liquid-biopsy analytes such as ctDNA). For clinical translation, regulatory and laboratory accreditation pathways for liquid-biopsy assays require fully specified pre-analytical conditions; the gaps identified here therefore represent a concrete barrier to advancing eccDNA from exploratory studies toward validated, tumor-context-specific clinical applications.

The translational value of eccDNA is likely to be greatest not in isolation but as one layer within an integrated, multi-omics liquid-biopsy framework. Circulating tumor DNA (ctDNA) and fragmentomic profiles capture point mutations, copy-number changes, and fragment-length signatures; DNA methylation and chromatin accessibility profiling add epigenetic and regulatory context; and exosomal RNA and circulating transcriptomic data report on active gene expression. eccDNA is mechanistically complementary to these layers because it frequently carries amplified oncogenes and can act as a mobile regulatory element, linking copy-number amplification to transcriptional output. Combining eccDNA with ctDNA, methylomic, transcriptomic, and single-cell readouts could therefore improve early detection, help resolve tumor heterogeneity, and enable longitudinal monitoring of treatment response and resistance [24]. Machine learning- and AI-assisted models are increasingly used to integrate such high-dimensional, multimodal data into composite biomarkers, and eccDNA junction and abundance features are well suited as inputs to these models [25]. Realizing this potential, however, depends on harmonized and transparently reported pre-analytical workflows because batch effects introduced upstream propagate and confound across data layers.

### 4.1. Limitations

This review also has several limitations. Firstly, the included studies were highly heterogeneous in the context of cancer type, sample size, biospecimen type, and study design. The studies investigated diverse malignancies with sample sizes varying from 6 to 252 participants. Some study designs involved healthy controls, but not all. While plasma and serum were the predominant biospecimen types investigated, some studies also used tissue or other types of bodily fluids such as bile or urine. Such heterogeneity limits comparability across studies and presents a barrier to assess reporting practices according to specific cancer types, biospecimen types, or study designs.

Secondly, because eccDNA remains an emerging biomarker in liquid biopsy research, only a small number of studies using plasma or serum are currently available. The limited sample size (n = 14) reduces the statistical power to detect consistent patterns across cancer types and hinders the robust evaluation of guideline adherence. Publication bias may also contribute to the limited range of eccDNA literature as studies reporting negative or statistically insignificant findings are less likely to be published than studies with conclusive and positive findings.

Thirdly, while most studies (13/14) included in our analysis were published after the release of the NCI guideline in 2020, the study by Kumar et al. [8] predates the guideline. As a result, it is important to be aware that the pre-analytical practices in that study should not be expected to reflect the guideline recommendations, which were not available at that time.

Finally, our analysis necessarily relied on published information; when studies did not report specific procedures, it remains unclear whether these steps were not performed or were simply omitted from the description. As a result, true adherence and deviation rates may differ from those captured in our review, raising additional concerns regarding transparency and reproducibility. Consequently, the findings of this review should be interpreted as an evaluation of reporting completeness in the recent clinical eccDNA literature rather than as evidence of a lack of methodological consistency or quality. Poor adherence to reporting recommendations does not directly correlate with poor experimental conduct or analysis. However, it does hinder the transparency, reproducibility, and comparability across eccDNA studies.

### 4.2. Existing Reporting and Standardization Initiatives

The need for better reporting methods in eccDNA research aligns with efforts to standardize biospecimen documentation and research reporting across biomedical fields. One important initiative is the Standard PREanalytical Code (SPREC), created by the International Society for Biological and Environmental Repositories (ISBER) in 2009 [26]. This code helps researchers document pre-analytical information that can affect the integrity of fluid and solid biospecimens [27]. SPREC aims to improve quality management and method harmonization by systematically coding variables related to biospecimen collection, processing, and storage that influence downstream analyses into a short string of letters [27]. After its release, SPREC has been integrated into biobank databases and quality management systems throughout the world, making it easier to standardize the annotation of pre-analytical biospecimens across different institutions [26]. Notably, many variables that this review found to be frequently underreported, such as blood collection procedures and freeze–thaw history, are factors that SPREC was designed to capture [26,27]. The continuous development of new versions of SPREC underscores the need to standardize comprehensive and concise documentation of pre-analytical variables in biospecimen-based research [28].

Another important effort comes from the EQUATOR (Enhancing the QUAlity and Transparency Of Health Research) Network. This network was established to improve the reliability of health research through more complete and clear reporting [29]. The EQUATOR Network has a searchable repository of reporting guidelines and encourages the development and use of reporting standards across a wide range of study designs and research fields [30]. While frameworks like CONSORT, STROBE, and PRISMA are widely used in clinical and observational studies, there is currently no reporting guideline specific to plasma- or serum-based eccDNA research. As a result, methodological reporting in eccDNA studies relies heavily on the practices of individual investigators, leading to the inconsistencies noted in this review.

### 4.3. Recommendations

The lack of detailed methodological reporting in eccDNA research highlights the urgent need for standardized reporting practices. To address this, we recommend the incorporation of a reporting checklist using the key pre-analytical variables described in the NCI guideline as a reference, encompassing 22 key items across biospecimen collection, blood processing, and eccDNA processing (Supplementary Table S3). The checklist includes the most crucial and frequently underreported pre-analytical procedural details for eccDNA studies, and it provides researchers with a succinct and easy-to-read way of reporting methodology. Examples of included items are whole-blood collection considerations (tube type, blood volume), as well as plasma/serum centrifugation information (speed, temperature, number of spins). Journals could encourage or require the submission of such a checklist as Supplementary Materials, similar to existing reporting guidelines used for systematic reviews. By ensuring that key pre-analytical details are transparently documented in publications, experimental reproducibility will be enhanced, and batch effects across studies will be reduced. This checklist may also be useful for biobanks that handle plasma or serum samples for eccDNA studies as a record for researchers to assess potential upstream sources of variability. Ultimately, improving methodological rigor and reporting standards will be essential for advancing eccDNA research toward clinical application in cancer diagnostics and monitoring. Our proposed checklist may provide a starting point for developing standardized reporting recommendations tailored specifically to eccDNA studies.

## 5. Conclusions

In conclusion, across 14 plasma/serum eccDNA studies, key pre-analytical and validation details were frequently underreported, while deviations from the NCI guideline, despite being rare, cannot be fairly assessed due to the frequency of underreporting. This issue indicates that the principal barrier lies in incomplete reporting. We recommend a concise eccDNA reporting checklist (Supplementary Table S3) with items informed by the pre-analytical details described in the NCI guideline. Inclusion of the checklist as a supplementary file during manuscript submission will effectively increase the transparency and consistency of methodological reporting across published studies. Biobanks are also encouraged to incorporate this checklist when releasing biospecimens to improve cross-institutional harmonization and transparency. Adoption by journals, biobanks, and other professional organizations in eccDNA research may facilitate more widespread efforts to standardize reporting guidelines. Ultimately, greater transparency in eccDNA research helps improve study reproducibility and accelerate the clinical translation of eccDNA as a biomarker for cancer detection, prognosis, and monitoring.

## Figures and Tables

**Table 1:** Summary of studies analyzed.

Author, Year, Reference	Type of Cancer Investigated	Sample Size	Type of Biospecimen Collected
Kumar et al., 2017 [8]	Lung cancer, ovarian cancer	12 lung cancer patients, 11 ovarian cancer patients	Serum/plasma
Li, J. et al., 2022 [9]	Colorectal cancer	89 patients with colorectal polyps, 163 colorectal cancer patients	Serum/plasma
Wu et al., 2022 [10]	Lung adenocarcinoma	6 patients	Plasma
Xu et al., 2022 [11]	Lung adenocarcinoma	23 patients, 13 normal controls	Serum/tissue
Bøllehuus Hansen et al., 2023 [12]	Pancreatic ductal adenocarcinoma	16 patients, 19 healthy controls	Plasma
Luo et al., 2023 [13]	Prostate cancer, hepatocellular carcinoma, colorectal cancer	2 prostate cancer patients, 12 hepatocellular carcinoma patients, 15 colorectal cancer patients, 4 healthy volunteers	Plasma/tissue
Fu et al., 2024 [14]	Perihilar cholangiocarcinoma	18 patients	Bile/plasma
Li, Q. et al., 2024 [15]	Clear cell renal cell carcinoma	3 patients, 3 healthy controls	Plasma
Yang et al., 2024 [16]	Lung cancer	25 patients, 6 healthy controls	Plasma/tissue
Zhou et al., 2024 [17]	Papillary thyroid carcinoma (PTC), nodular thyroid goiter (NOD)	47 PTC patients, 25 NOD patients, 13 normal thyroid volunteers	Plasma/tissue
Zole et al., 2024 [18]	Lung adenocarcinoma	4 patients, 4 healthy donors	Plasma
Chen et al., 2025 [19]	Multiple myeloma	98 patients, 10 healthy donors	Serum
Lu et al., 2025 [20]	Breast cancer	20 patients, 15 healthy volunteers	Plasma/tissue
Zhang et al., 2025 [21]	Prostate cancer	49 prostate cancer patients, 23 patients with prostatitis, 21 healthy controls	Plasma/urine/tissue

## Data Availability

The original contributions presented in this study are included in the article/Supplementary Materials. Further inquiries can be directed to the corresponding authors.

## Supplementary Materials

The following supporting information can be downloaded at https://www.mdpi.com/article/10.3390/cancers18142196/s1, Figure S1: Deviation rate of practice recommended by the NCI guideline; Table S1: Item-level checklist for elements reported, missing, or deviated from the recommended practice; Table S2: PRISMA checklist for the systematic review [31]; Table S3: Standardized method reporting checklist for human plasma/serum eccDNA research.
