# Supplementary material for: Assessing Reporting Quality and Pre-Analytical Standards in Extrachromosomal Circular DNA Studies in Cancer: A Systematic Review
Source: Cancers (Basel). 2026 Jul 8;18(14):2196. doi: 10.3390/cancers18142196 (PMC13406781; doi:10.3390/cancers18142196)
Supplement: Supplementary file 1 [file cancers-18-02196-s001.zip › Supplementary Table S3.pdf]

Standardized Method Reporting Checklist for Human Plasma/Serum eccDNA Research

A) Study information and Biospecimen Collection

Sample size			Biospecimen type		
**Patient information**					
Age		Gender		Diagnosis	
Fasting status		Treatment status		Time of last treatment	
**Collection considerations**					
Collection tube type			Blood volume		
**Blood collection process**					
Date of blood collection		Venipuncture site		Type of blood collection needle	

B) Blood Processing

**Blood processing delay**					
**Plasma/serum separation by centrifugation**					
Speed		Temperature		Number of spins	
**Interim storage**					
Storage duration		Temperature		Number of freeze-thaw cycles	

C) eccDNA Processing

**Extraction procedure**	
**Quantification methods**	
**Evaluation of suitability for subsequent analysis**	
**Validation of analytical assay**	
