# Supplementary figures and images for: Assessing Reporting Quality and Pre-Analytical Standards in Extrachromosomal Circular DNA Studies in Cancer: A Systematic Review
Source: Cancers (Basel). 2026 Jul 8;18(14):2196. doi: 10.3390/cancers18142196 (PMC13406781; doi:10.3390/cancers18142196)

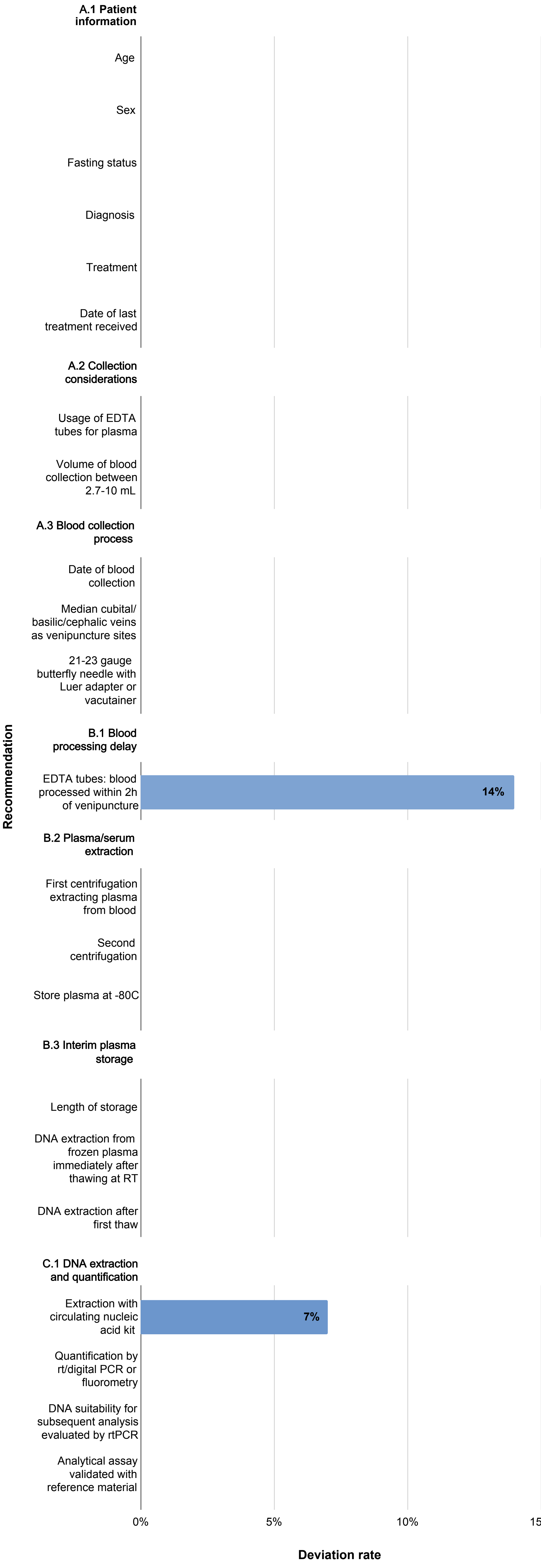

Supplement: Supplementary file 1 [file cancers-18-02196-s001.zip › Supplementary_Figure S1.pdf]
